# Failure rate and complications of small-bore, wire-guided chest drains in adult patients presenting with traumatic and nontraumatic pleural diseases: A systematic review

**DOI:** 10.5339/qmj.2025.55

**Published:** 2025-06-09

**Authors:** Zubaidah Alomar, Zainab Tawfek, Yousif Alomar, Ismail Mahmood, Ali Alomar, Ayman El-Menyar, Sandro Rizoli, Hassan Al-Thani

**Affiliations:** 1 Jordan University of Science and Technology, Irbid, Jordan; 2 Emergency Department, Al Ahli Hospital, Doha, Qatar; 3 Trauma Surgery, Hamad Medical Corporation, Doha, Qatar; 4 Surgery Department, Qatar University, Doha, Qatar; 5 Clinical Medicine, Weill Cornell Medicine, Doha, Qatar *Email: aymanco65@yahoo.com

**Keywords:** Small-bore catheter, small-bore chest drain, pigtail, pleural diseases, chest, thorax, failure rate, complications

## Abstract

**Background:**

Pleural diseases are common and often require drainage, with the growing use of small-bore chest drains (SBCDs) instead of larger tubes. This review aimed to examine the failure rate and complications associated with SBCD use in different pleural pathologies.

**Methods:**

A literature search (PubMed, SCOPUS, and Google Scholar) was performed on the complications associated with SBCDs to treat pleural diseases. This review analyzed patient demographics, indications, outcomes, failure rate, and complications associated with the use of SBCDs. The systematic review was conducted using PRISMA (Preferred Reporting Items for Systematic reviews and Meta-Analyses) guidelines.

**Results:**

Thirty studies were included in this review with 4,973 patients. The indications for insertions of SBCDs were pleural effusion at 48.4%, pneumothorax at 30.1%, empyema or parapneumonic effusion at 11.4%, hemothorax at 6.5%, and other indications at 3.6%. The overall failure rate to achieve satisfactory drainage was 19.4%. Significant complications included iatrogenic pneumothorax at 11.9%, major hemorrhage at 1.0%, local bleeding at 0.7%, infection at 1.2%, and iatrogenic organ injury at 0.9%. Other insertional complications included tube dislodgement at 5.9%, tube blockage at 5.4%, tube kinking at 3.7%, misplacement at 3.3%, and subcutaneous hematoma at 0.5%. Most of the data published revolves around hemodynamically stable patients with SBCD insertions and is, thus, deficient regarding hemodynamically unstable patients.

**Conclusion:**

Despite carrying notable failure rates and complications, small-bore catheters remain an acceptable option for managing selected pleural diseases.

## Introduction

Insertion of a tube in the pleural cavity is a common medical procedure used to manage conditions affecting the pleura, such as the accumulation of air, blood, or fluid. It facilitates lung reexpansion and helps prevent complications. It is indicated for conditions such as pneumothorax, hemothorax, empyema, and postsurgical pleural effusions, while contraindications include severe coagulopathy, extensive pleural adhesions, and infected skin at the insertion site.^1,2^

These tubes are made from materials like polyvinyl chloride, polyethylene, or silicone and are available in straight, angled, or coiled (pigtail) designs. Their size, measured in French (Fr), ranges from 8 Fr to 36 Fr, with small-bore (8–14 Fr) and large-bore (>14 Fr) classifications. One French unit equals one-third of a millimeter, meaning a 12 Fr tube has a 4 mm diameter.^1^

Small-bore catheters have emerged as a reliable and more comfortable alternative to traditional chest tubes, offering a smaller insertion site for draining various thoracic conditions such as pleural effusion, pneumothorax, hemothorax, and empyema.^2^ One advantage of small-bore wire-guided drains is their ability to be placed at the patient’s bedside, making them particularly useful in critical care settings. In contrast, large-bore drains, due to their more invasive nature and larger size, often require sedation or, in some cases, general anesthesia for insertion in a surgical setting.^1^ Unlike traditional tube thoracostomy, small-bore catheters feature a smaller diameter, increase flexibility, and cause less tissue damage.^3^ However, their invasive nature and narrower size make them more prone to obstruction, kinking, and iatrogenic organ damage.^4^ Over time, advances in medicine have started shifting to favor small-bore chest drains (SBCDs) as the preferred method of treatment for lung diseases.^1^ The aim of this systematic review is to explore the failure rate and complications after SBCD use and to address the indications and failure rate.

## Methods

**Figure 1:**
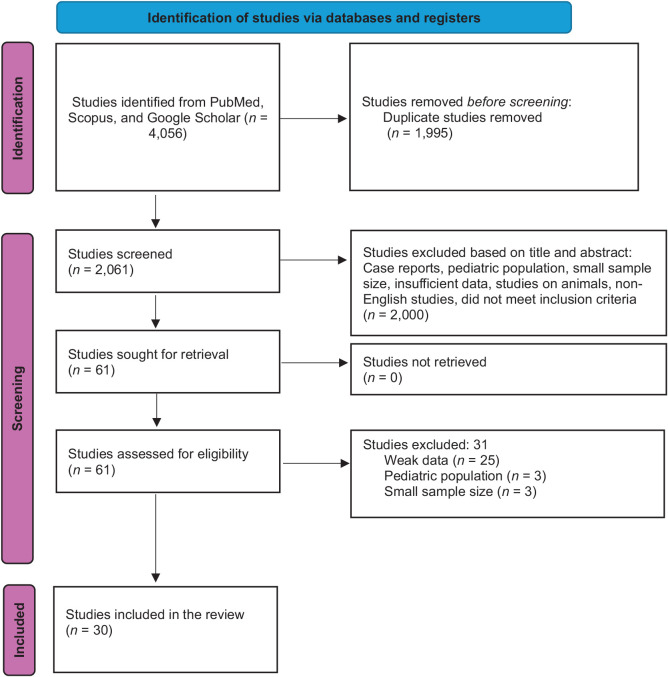
PRISMA flow diagram showing study selection.

This review was performed according to the PRISMA (Preferred Reporting Items for Systematic reviews and Meta-Analyses) guidelines. A systematic literature search (PubMed, SCOPUS, and Google Scholar) describing the use of small-bore catheters in pleural diseases was performed from database inception to February 7, 2024. The following search keywords were used: “small bore” OR “pigtail” AND “chest” OR “pleural” OR “thorax” AND “complications.” Additionally, systematic reviews covering similar topics were screened for additional primary studies that had not yet been included. Likewise, the lists of references for the included studies were scanned for potential further studies that would have been overlooked. The search identified 2,061 records, 61 of which met the inclusion criteria based on their title and abstract. Further studies were then excluded following the detailed reading of the complete text, leaving 30 studies deemed eligible. The search process is illustrated in Figure 1.

Exclusion criteria were as follows: pediatrics, case reports, nonlung cases, failure to report complications, weak data, less than 20 patients, articles published before 2000, articles published in languages other than English, studies that did not include at least two of the following areas of interest: indications, failure rate, complications, hospital length of stay (LOS), and duration of drainage.

A single author did the literature search and then was cross-checked by another author. Data was extracted by one author and double-checked by another. If any disagreements arose, a third party was consulted to resolve the issue. The list of data to be extracted was as follows: study type, number of patients, year published, catheter size, insertion technique, mean age, incidence in males and females, duration of drainage, hospital LOS, injury severity score, use of image guidance, indications for insertion, failure rate, secondary procedure (if applicable), major complications and tube related complications. While retrieving the indications, we sought to distinguish between spontaneous (primary and secondary) and traumatic pneumo-thoraces and malignant and nonmalignant effusions. Failure was defined as the need for further intervention, such as the insertion of a second catheter or a large-bore chest tube, thoracostomy, or video-assisted thoracoscopic surgery (VATS). Any discrepancies were resolved by collaboration and discussion.

The included studies were assessed for quality of evidence using the GRADE (Grading of Recommendations Assessment, Development and Evaluation) criteria.^5^ Overall, the quality of evidence ranged from moderate to high (Table 1). The Newcastle-Ottawa Scale (NOS) (Table 2) was used to assess the risk of bias in retrospective and prospective cohort studies, while randomized controlled trials (RCTs) were evaluated using the Cochrane Risk of Bias 2 (RoB 2) tool (Table 3) for randomized trials. Among the studies, 25 had a low risk of bias,^8,10,12–17,19–35^ while 5 showed a moderate risk of bias.^6,7,9,11,18^

## Results

A comprehensive literature search initially yielded 4,056 articles. Following the removal of duplicates and subsequent screening and full-text reviews, 30 relevant studies were identified (Figure 1). These studies included three RCTs, seven prospective studies, 1 case control, and 19 retrospective studies. A total of 4,973 patients (5,338 SBCD insertions) were enrolled in this study. The percentage of male patients was 64.3%, the mean age of patients was 51.1 years, and the mean hospital LOS was 13.8 days. The average drain catheter size was 12 Fr (range 7–16 Fr), and the average duration of drainage was 4.7 days. Regarding the insertion technique, 27 studies utilized the Seldinger technique, which had an overall failure rate of 19.4%, ranging from 4.2% to 42%. One study^29^ exclusively used the trocar technique, reporting a failure rate of 29.8%. Additionally, two studies^8,33^ incorporated both the trocar and Seldinger techniques, with failure rates of 15.4% and 12.0%, respectively. Furthermore, 13 out of the 30 studies used image guidance to insert the catheter. Table 4 summarizes the characteristics of the studies as well as their associated patient demographics.

Eighteen studies reported that insertions were performed by attending physicians, radiologists, or residents under attending supervision.^8–15,19,22–24,26,27,29,31,33,35^ Eleven studies did not specify the level of the performing physician.^6,16–18,20,21,25,28,30,32,34^ Only one study documented that insertions were performed by residents without supervision.^7^ Although the study concluded that small-bore pleural drain placement by residents is a safe procedure, it was associated with a high incidence of irrelevant pneumothoraces. The author suggested that to improve patient safety, resident-performed procedures should be supervised by a senior physician.^7^

**Figure 2:**
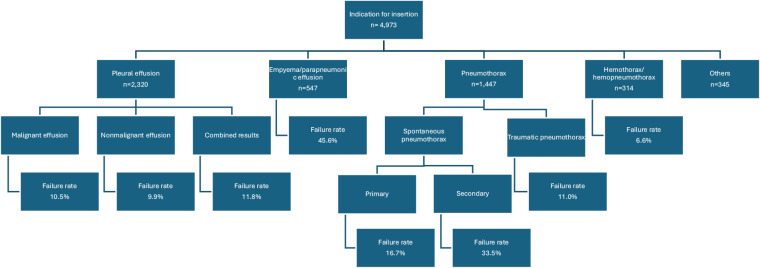
Indications for insertion of small-bore catheter and their failure rates.

There were four primary indications for the insertion of an SBCD: pleural effusion, pneumothorax, hemothorax, and empyema (Figure 2 and Table 5). Pleural effusion was the most frequent reason, representing 2,320 patients. 15 studies^6,8,9,13,17,20,22,24,26–28,32–35^ specified whether the pleural effusions were malignant or nonmalignant, whereas one study^11^ did not provide information on the cause of the pleural effusions. Overall, 58.5% of pleural effusions were malignant, 27.0% were nonmalignant, and 14.5% were unclassified. Moreover, the failure rate for all types of pleural effusions was 9.7%.

Pneumothorax was the second most frequent indication, with a total of 1,447 cases. Among these, 1,035 cases (72.4%) were identified as spontaneous pneumothorax.^14,16,17,19,25,29^ Among the spontaneous pneumothorax cases, 57.4% were primary, while the rest were secondary. Additionally, 10.7% were attributed to traumatic pneumothorax.^9,10,15^ Five studies, representing 17.8% of the patients, did not specify whether the pneumothorax was traumatic or spontaneous.^6,13,27,33,35^

The overall failure rate for managing pneumothorax was 22.4%. The average failure rate for primary spontaneous pneumothorax was 16.7%, while for secondary spontaneous pneumothorax it was 33.5%. For uncomplicated traumatic nonemergent pneumothorax, the failure rate was 11.0%.

The third most common indication was empyema or parapneumonic effusion, comprising 547 cases. It also had the highest failure rate among all indications, 45.6%. Six studies^13,17,22,24,26,33^ reported failure rates of empyema, ranging from 9.0% to 80.0%.

Finally, the least common indication was hemothorax or hemopneumothorax, accounting for 314 patients.^9,12,13,15,26,27,30,31^ The circumstances for the insertion of SBCDs were stable, nonemergent hemothorax or hemopneumothorax, with an average failure rate of 6.6%.

All in all, the failure rate for thoracic diseases undergoing small-bore catheter insertions was 19.4% in achieving satisfactory drainage (Table 6). Among these patients, further interventions were required: 5.1% needed a second catheter insertion, 3.2% required a large-bore chest tube insertion, 1.0% needed VATS, and 0.9% required thoracostomy. There were no reported deaths directly resulting from these complications.

**Figure 3:**
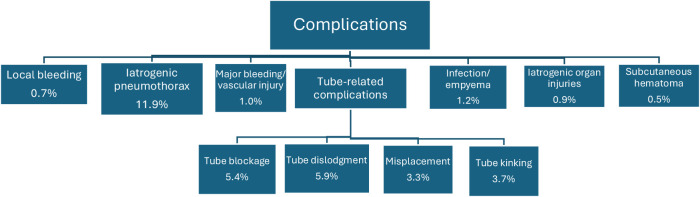
Complication rates of the small-bore catheter.

Complications included iatrogenic pneumothorax (not present prior to procedure) 11.9%, major hemorrhage 1.0%, local bleeding 0.7%, empyema or infection 1.2%, and iatrogenic organ injury 0.9% (Figure 3 and Table 7). Examples of the iatrogenic injuries that occurred were intercostal artery injury, lung transection, subclavian vein insertion, diaphragmatic penetration, intrahepatic placement, and spleen perforation. Additionally, there was a rare case of an epidural malposition^11^ where the catheter pierced through an intervertebral foramen into the subarachnoid space, resulting in liquor drainage without any neurological sequelae.

Regarding comorbidities, 28 studies did not report them or establish a correlation with complications. One study identified comorbidities such as advanced age, hypertension, diabetes, and smoking history as significantly increasing the incidence of complications.^27^ Additionally, another study linked three infectious complications to immunocompromised conditions.^14^

Catheter-related insertion complications included tube dislodgement (5.9%), tube blockage (5.4%), tube kinking (3.7%), misplacement (3.3%), and subcutaneous hematoma (0.5%) (Table 8). In examining the correlation between image guidance and insertion complication rates, 13 studies used image guidance, while 17 studies did not. The median complication rate for image-guided insertions is 6.45%, with a range from 1.6% to 77.5%. For non-image-guided insertions, the median complication rate is 8.15%, ranging from 0.4% to 36%.

Other less common complications include subcutaneous emphysema, vasovagal episode, hydrothorax, reexpansion pulmonary edema, and incisional site infection.

## Discussion

There is a rising trend toward using smaller-diameter chest drains, which have now become standard practice in many healthcare facilities.^1^ This shift in practice has occurred despite the limited number of well-conducted RCTs that demonstrate the efficacy and complications associated with small-bore tubes. However, some retrospective and prospective observational studies suggest that smaller-bore drains are less painful compared to larger (>24 Fr) caliber thoracostomy tubes.^2^

Pleural effusion was the most frequent indication in our review, accounting for nearly half of the cases at 48.4%. It is a common complication in intensive care unit (ICU) patients,^36^ with failure rates ranging from 7.7% to 62%. Large pleural effusions can significantly affect the cardiorespiratory system, leading to abnormalities in gas exchange, respiratory mechanics, muscle function, and hemodynamics. While the risk-benefit ratio of pleural drainage in critically ill patients is not fully established, draining a pleural effusion may impact patient outcomes and diagnostic accuracy.^37,38^

Pleural effusions were classified into malignant and nonmalignant categories. Nonmalignant causes include congestive cardiac failure, cirrhosis, parapneumonic effusion, tuberculosis, pleuritis, hepatic hydrothorax, postcoronary artery bypass surgery, bile leak, peritoneal dialysis, trauma, chronic renal failure, or undiagnosed cases.^17,22^ Malignant effusions are associated with lung cancer, mesothelioma, genitourinary tract neoplasms, hematologic neoplasms, and gastroenteric neoplasms.^17^ The failure rate for all types of effusions ranged from 6.2% to 27.7%, with an average of 9.7%. Four studies reported failure rates between 6.2% and 24.5% for malignant effusions, with an average of 10.5%.^14,18,25,29^ Variations in these failure rates may be influenced by the disease’s progression and the effectiveness of treatment.

Pneumothorax was the second most common indication for SBCDs. It was categorized into spontaneous and traumatic types. Spontaneous pneumothorax is further divided into primary and secondary. Primary spontaneous pneumothorax occurs in individuals without preexisting lung disease, though they may have had asymptomatic bullae or blebs that burst. In contrast, secondary spontaneous pneumothorax arises in the context of existing lung conditions, such as chronic obstructive pulmonary disease, tuberculosis, or sarcoidosis.^39^

The highest failure rate was observed in secondary spontaneous pneumothorax, at 33.5%, with a range from 29.8% to 52.6% [29, 16, respectively]. The next highest failure rate was for primary spontaneous pneumothorax, which averaged 16.7%, ranging from 15.5% to 40% [19, 24, respectively]. Finally, there were only a small number of trauma patients, with a failure rate of 11.0% for uncomplicated traumatic nonemergent pneumothorax, ranging from 5.0% to 13.3% [31, 15, respectively].

Similarly, there is limited data on traumatic hemothorax and hemopneumothorax. The failure rates ranged from 5.0% to 33.3%, with an average failure rate of 6.6%. The studies were conducted in the context of stable, nonemergent hemothorax and excluded cases involving emergency catheter placement due to hemodynamic instability. Furthermore, the studies did not include details on the volume of hemothorax observed on CT scans, whether mild or moderate, before the tube was inserted. However, the initial output was reported, which ranged from 600 ml to 810 ml.^12,30,31^ Moreover, the tube was inserted in a delayed, stable setting, typically between 2 and 4.5 days after the injury. At this late stage, it is impossible to distinguish whether the condition is due to an expansion of hemothorax, a pleural effusion resulting from fluid resuscitation, other effusions related to major solid organ injuries below the diaphragm, or a combination of these factors.

Empyema is a prevalent medical condition with a mortality rate of up to 20%.^40^ Failure rates in treating empyema range from 9% to 80%. Compared to simpler fluids, the high viscosity of pus causes it to flow more slowly through smaller tubes. Therefore, it is preferable to use large-bore chest catheters in this situation compared to other indications such as simple effusion or pneumothorax.^41^ Moreover, multilocular empyema can make drainage more challenging. Additionally, fibrinous material can obstruct the drainage holes at the tip of the catheter. Flushing the drains with saline solution may help reduce the likelihood of such blockages.^42^ Intrapleural fibrinolytic therapy was linked to a decreased need for surgical intervention and overall treatment failure, but there was no evidence of a change in mortality rates. However, this approach could result in pleural hemorrhage and systemic hemorrhage.^42^

In general, while many of the studies do not explicitly mention the use of antibiotic prophylaxis, it is standard practice in clinical settings to administer antibiotics to prevent infection. Three authors did not routinely use antibiotic prophylaxis before insertion,^12,15,31^ and 1 study gave antibiotics to all patients.^17^ Antibiotic use should be tailored based on individual patient risk factors, but not routinely for all cases.^6,9^

A few serious complications have arisen from the insertion of Seldinger’s SBCDs. These complications include major hemorrhage from chest wall vessels or lung parenchyma, as well as injuries to the liver and spleen.^21^ Though rare, a disastrous complication that can occur is heart perforation.^43^

One method to minimize complications is to use imaging guidance during insertion. However, this approach can increase costs and cause delays in many healthcare facilities due to the requirement for radiologists’ involvement. It is also important to note that both blind and image-guided drain insertions can lead to patient morbidity.^6^ A recent meta-analysis including seven studies assessed the failure rate of drainage tubes (use of second tube placement or VATS, unresolved pneumothorax, hemothorax, or hemopneumothorax) and the initial drainage output, ICU LOS, and ventilator days in trauma.^44^ The authors found that pigtail had higher initial output volumes vs chest tube use. Moreover, the chest tube group had a higher risk of requiring VATS vs the pigtail group.

This systematic review presents several limitations. Most of the included studies are retrospective, so complications and outcomes may have been underestimated because of potential documentation failures or underreporting. Additionally, the comprehensive nature of our review highlights heterogeneity in the indications across different diseases, and meta-analysis was not conducted. Regarding pleural effusions, there were no standardized criteria for tube insertion, and most studies did not document the volume of pleural effusion.

Moreover, decisions for insertion were based on physician discretion, preference, and chest X-ray findings rather than on volumetric assessments using CT scans or ultrasound. Furthermore, there is not enough data to draw conclusions regarding the usage of SBCDs to manage pneumothorax, hemothorax, and hemopneumothorax in acute emergency settings. This gap in evidence highlights the need for additional research to clarify the role of small-bore catheters in managing thoracic-related trauma and other medical conditions, which could eventually lead to improved treatment guidelines and optimal patient care.

## Conclusion

The systematic review reveals a wide range of failure rates for tube insertion, accompanied by notable morbidity. Although the procedure is deemed effective for many clinical conditions, outcomes are significantly influenced by the procedure’s indication and the type of pleural disease. Further research is necessary to clarify the indications, benefits, and risks of using small-bore catheters for the management of traumatic and nontraumatic thoracic disease.

## Authors’ contribution

ZA, YA, and IM: conceptualization; ZA, YA, ZT, and IM: methodology; ZA, YA, and IM: formal analysis; ZA, YA, ZT, and IM: data curation and writing—original draft preparation; AE, HA, and SR: writing—review and editing. All authors have read and agreed to the published version of the manuscript.

## Conflict of interests

The authors declare no conflicts of interest.

## Data availability statement

All data were presented in the manuscript and tables.

## Conference presentation

This review was presented partly at the 3rd Euro Anesthesiology and Critical Care Congress (EACCM 2024), October 28–29, 2024, London, UK.

## Figures and Tables

**Table 1.** Quality of evidence assessment utilizing the GRADE criteria.

**Study**	**Study design**	**Risk of bias**	**Inconsistency**	**Indirectness**	**Imprecision**	**Publication bias**	**Overall quality of evidence (GRADE)**
Davies et al.^6^	Cohort study	Moderate (due to lack of control group)	None	None	Moderate (sample size not specified)	None	Moderate
Vetrugno et al.^7^	Cohort study	Moderate (lack of control group and nonrespondent info)	None	Moderate (ICU patients, may not generalize)	Moderate (no statistical analysis provided)	None	Moderate
Congedo et al.^8^	Cohort study	Moderate (lack of control for confounders)	None	None	Moderate (potential confounding factors not controlled)	None	Moderate
Kulvatunyou et al.^9^	Cohort study	Moderate (no control for confounders)	None	Moderate (focused on trauma patients)	Moderate (no clear follow-up data)	None	Moderate
Kulvatunyou et al. ^10^	RCT	Low (randomization, proper control)	None	Low (directly compares pigtail catheters vs chest tubes for traumatic pneumothorax)	Moderate (sample size could be larger)	None	High
Treml et al.^11^	Cohort study	Moderate (no control for confounders)	None	None	Moderate (no statistical analysis provided)	None	Moderate
Kulvatunyou et al.^12^	RCT	Low (randomization, proper control)	None	Low (focuses on traumatic hemothorax, directly applicable)	Moderate (small sample size)	None	High
Liu et al.^13^	Cohort study	Moderate (lack of control group, no randomization)	None	Moderate (results may not apply to all pleural diseases)	Moderate (small sample size, no statistical analysis)	None	Moderate
Lin et al.^14^	Cohort study	Moderate (no randomization, potential confounding)	None	Moderate (limited to mechanically ventilated patients)	Moderate (small sample size, limited statistical analysis)	None	Moderate
Rivera et al.^15^	Cohort study	Moderate (no randomization or control for confounding)	None	Moderate (focuses on trauma patients, limiting generalizability)	Moderate (small sample size, lack of statistical analysis)	None	Moderate
Cho and Lee^16^	Cohort study	Moderate (no control for confounding variables)	None	Moderate (limited generalizability to broader pneumothorax cases)	Moderate (small sample size, limited statistical analysis)	None	Moderate
Cafarotti et al.^17^	Cohort study	Moderate (no randomization, some confounding)	None	Moderate (focus on specific diseases may limit applicability)	Moderate (limited sample size, lack of statistical rigor)	None	Moderate
Messa et al.^18^	Cohort study	Moderate (no control for confounding, retrospective design)	None	Moderate (focus on trauma and thoracic trauma treatment)	Moderate (no clear follow-up data or statistical analysis)	None	Moderate
Salé et al.^19^	Cohort study	Moderate (potential for confounding factors in multicenter study)	None	Moderate (restricted to ambulatory management of pneumothorax)	Moderate (sample size not large enough to ensure statistical significance)	None	Moderate
Li et al.^20^	Cohort study	Moderate (no randomization or blinding)	None	Moderate (limited to postoperative lung cancer patients)	Moderate (small sample size, limited statistical details)	None	Moderate
Bauman et al.^21^	Cohort study	Moderate (no randomization, potential confounding factors)	None	Moderate (focus on trauma patients, may not apply to other populations)	Moderate (no clarity on long-term follow-up or complications)	None	Moderate
Keeling et al.^22^	Cohort study	Moderate (no randomization, potential bias)	None	Moderate (focus on radiology-guided catheter drainage)	Moderate (small sample size, lack of statistical analysis)	None	Moderate
Kulvatunyou et al.^23^	Cohort study	Moderate (no randomization, potential for confounding)	None	Moderate (focused on trauma patients)	Moderate (limited sample size, no detailed statistical analysis)	None	Moderate
Horsley et al.^24^	Cohort study	Moderate (no randomization, risk of confounding)	None	Moderate (specific to small-bore chest tubes, not generalizable to all pleural procedures)	Moderate (limited statistical analysis, small sample size)	None	Moderate
Vedam and Barnes ^25^	Cohort study	Moderate (no control for confounding, retrospective)	None	Moderate (focus on pneumothorax, not applicable to all pleural conditions)	Moderate (small sample size, lack of statistical rigor)	None	Moderate
Liang et al.^26^	Cohort study	Moderate (no randomization, potential for confounding)	None	Moderate (restricted to ICU patients, limiting generalizability)	Moderate (small sample size, lack of statistical analysis)	None	Moderate
Mortman et al.^27^	Cohort study	Moderate (no randomization, retrospective)	None	Moderate (compares pigtail catheters to surgical chest tubes, not generalizable to all scenarios)	Moderate (small sample size, limited statistical analysis)	None	Moderate
Jayakrishnan et al.^28^	Cohort study	Moderate (potential confounding)	None	Moderate (specific to pleural effusion treatment in Oman, limited external validity)	Moderate (small sample size, limited analysis)	None	Moderate
Chen et al.^29^	Cohort study	Moderate (no randomization or blinding, potential for confounding)	None	Moderate (secondary pneumothorax cases, not fully generalizable)	Moderate (sample size too small for precision)	None	Moderate
Orlando et al.^30^	Cohort study	Moderate (retrospective, potential for bias)	None	Moderate (focus on delayed hemothorax, may not be relevant to all cases)	Moderate (small sample size, lack of follow-up data)	None	Moderate
Bauman et al.^31^	RCT	Low (randomization, appropriate control)	None	Low (direct comparison of chest tubes vs pigtail catheters for hemothorax and hemopneumothorax)	Moderate (sample size could be larger)	None	High
Rafiq et al.^32^	Cohort study	Moderate (retrospective, risk of confounding)	None	Moderate (focuses on loculated pleural effusion, limited generalizability)	Moderate (limited sample size, statistical uncertainty)	None	Moderate
Cantin et al.^33^	Cohort study	Moderate (no randomization, potential bias)	None	Moderate (single-center study, limits external validity)	Moderate (small sample size, limited statistical rigor)	None	Moderate
Jain et al.^34^	Cohort study	Moderate (no randomization, risk of confounding)	None	Moderate (focused on military population, not widely applicable)	Moderate (small sample size, lack of statistical rigor)	None	Moderate
Tajarernmuang et al.^35^	Cohort study	Moderate (retrospective, risk of confounding)	None	Moderate (focused on practice review, not randomized)	Moderate (small sample size, no follow-up data)	None	Moderate

**Table 2.** Risk of bias assessment for cohort studies using the NOS.

**Author**	**Selection**	**Comparability**	**Outcome**	**Quality score (out of 9)**
Davies et al.^6^	3	0	3	6
Vetrugno et al.^7^	2	0	2	4
Congedo et al.^8^	4	0	3	7
Kulvatunyou et al.^9^	2	0	3	5
Treml et al.^11^	3	1	2	6
Liu et al.^13^	3	2	2	7
Lin et al.^14^	3	2	2	7
Rivera et al.^15^	4	2	3	9
Cho and Lee^16^	3	2	2	7
Cafarotti et al.^17^	4	2	3	9
Messa et al.^18^	3	1	2	6
Salé et al.^19^	4	2	3	9
Li et al.^20^	3	2	3	8
Bauman et al.^21^	4	2	3	9
Keeling et al.^22^	3	2	3	8
Kulvatunyou et al.^23^	4	2	3	9
Horsley et al.^24^	4	2	3	9
Vedam and Barnes^25^	4	2	3	9
Liang et al.^26^	4	2	3	9
Mortman et al.^27^	3	2	3	8
Jayakrishnan et al.^28^	3	2	3	8
Chen et al.^29^	3	2	3	8
Orlando et al.^30^	4	2	3	9
Rafiq et al.^32^	3	2	3	8
Cantin et al.^33^	3	1	3	7
Jain et al.^34^	3	1	3	7
Tajarernmuang et al.^35^	3	1	3	7

**Table 3.** Risk of bias for RCTs using the Cochrane Risk of Bias 2 (RoB 2) tool.

**Authors**	**Random sequence generation**	**Allocation concealment**	**Blinding of participants and personnel**	**Blinding of outcome assessment**	**Incomplete outcome data**	**Selective reporting**	**Other bias**
Kulvatunyou et al.^10^	Low risk	Low risk	Low risk	Low risk	Low risk	Low risk	Low risk
Kulvatunyou et al.^12^	Low risk	Low risk	Low risk	Low risk	Low risk	High risk	Low risk
Bauman et al.^31^	Low risk	Low risk	Low risk	Low risk	Low risk	Low risk	Low risk

**Table 4.** Patient demographics.

	**Year published**	**Study type**	**Number of patients (number of insertions)**	**Drain catheter size (Fr)**	**Insertion technique**	**Mean age**	**Male (%)**	**Duration of drainage (days)**	**Mean hospital LOS in (days)**	**ISS**	**Image-guided**
HE Davies et al.	2007	Retrospective	91 (100)	<14	Seldinger	62	59	2.6	nr	nr	Yes
Vetrugno et al.	2019	Retrospective	71 (87)	8–14	Seldinger	69.48 ±11.55	57.7	nr	nr	nr	Yes
MT Congedo et al.	2023	Prospective	117	12	Trocar, Seldinger	63.53 ± 14.82	20.8	nr	nr	nr	No
Kulvatunyou et al.	2011	Retrospective	94	14	Seldinger	43 ± 21	64	4 ± 1.6	6	17 ±11	No
Kulvatunyou et al.	2014	RCT	20	14	Seldinger	46	85	2	4	14.5	No
Treml et al.	2021	Retrospective	247 (396)	10	nr	71	66.8	nr	nr	nr	No
Kulvatunyou et al.	2021	RCT	56	14	Seldinger	56 ± 17	84	4	8.5	17.8 ± 6.8	Yes
Y-H Liu et al.	2009	Retrospective	276 (332)	10–16	Seldinger	59 ± 18	64.5	6.1 ± 2	29.23 ± 29.6	nr	Yes
Y-C Lin et al.	2009	Retrospective	62 (70)	12–16	Seldinger	63.8 ± 20.3	66	5.9	36.7	nr	Yes
Rivera et al.	2008	Retrospective	107 (131)	10–14	Seldinger	49.8	50.3	5.5	14.2	19.1	Yes
S Cho et al.	2010	Retrospective	200	7	nr	37.2 ± 21.8	86	nr	nr	nr	No
Cafarotti et al.	2010	Retrospective	1092	12	Seldinger	56 ± 18	38.7	5.35	nr	nr	No
Messa et al.	2021	Retrospective	44	≤19	nr	50.0	86	3.27 ± 2.46	14.8	14.8	No
A Sale et al.	2020	Prospective	148	8.5	nr	31	74.3	nr	6.1	nr	No
Xianshuai Li et al.	2022	Case control	677	8	nr	59.2 ± 14.1	41.5	nr	nr	nr	No
Bauman et al.	2017	Prospective	189	14	Seldinger	52 ± 21	76	4	7	16	No
Keeling et al.	2007	Retrospective	82 (93)	8–12	nr	66	61	nr	nr	nr	Yes
Kulvatunyou et al.	2012	Prospective	36	14	Seldinger	53 ± 4	75	5 ± 0.8	10	18 ± 1.5	No
Horsley et al.	2006	Prospective	44 (52)	12–20	Seldinger	64 ± 2	nr	4.5±0.5	nr	nr	No
Vedam et al.	2002	Retrospective	36	9	nr	37	nr	nr	5	nr	No
Liang et al.	2008	Retrospective	133 (142)	10–14	Seldinger	63.7 ± 15.4	70	nr	nr	nr	Yes
Mortman et al.	2023	Retrospective	326	≤14	nr	58 ± 18	64	5.9 ± 7.5	20 ± 26	nr	Yes
B. Jayakrishnan et al.	2020	Retrospective	141	8.5–14	Seldinger	53.9	52.5	5	nr	nr	Yes
C-H Chen et al.	2010	Retrospective	168	10–16	Trocar	60.3 ± 18.3	86	6.9 ± 4.3	16.0 ± 16.8	nr	Yes
Orlando et al.	2020	Retrospective cohort	160 (191)	12	nr	44.2	70	3.6	5.1	17.8	No
Bauman et al.	2020	RCT	20	14	Seldinger	62 ± 13	85	4	6.5	17.5 ± 6.6	No
Rafiq et al.	2020	Prospective	30	10	Seldinger	44	57	6	nr	nr	Yes
Cantin et al.	2005	Retrospective	51	6–12	Trocar, Seldinger	58.4	37	7	48	nr	Yes
Jain et al.	2006	Prospective	50	9–12	Seldinger	nr	nr	7	nr	nr	No
Tajarernmuang et al.	2021	Retrospective	205 (249)	8–12	Seldinger	nr	59	3.5	21.5	nr	No

nr: not reported.

**Table 5.** Indications of chest drain.

**Article**	**Number of patients (number of insertions)**	**Reasons for insertion**						
		**Pneumothorax**	**Empyema/Parapneumonic effusion**	**Malignant effusion**	**Nonmalignant effusion**	**Hemothorax/Hemopneumothorax**	**Unreported type of pleural effusion**	**Other**
HE Davies et al.	91 (100)	21	18	42	10	nr	nr	nr
Vetrugno et al.	71 (87)	nr	nr	nr	nr	nr	nr	nr
MT Congedo et al.	117	nr	4	86	nr	nr	24	3
Kulvatunyou et al.	94	75TR	nr	nr	8	9TR	nr	2
Kulvatunyou et al.	20	20TR	nr	nr	nr	nr	nr	nr
Treml et al.	247 (396)	nr	nr	nr	nr	nr	247	nr
Kulvatunyou et al.	56	nr	nr	nr	nr	56TR	nr	nr
Y-H Liu et al.	(332)	64	119	98	nr	9	38	4
Y-C Lin et al.	(70)	70SEC	nr	nr	nr	nr	nr	nr
Rivera et al.	107 (131)	60TR	1	nr	41	36TR	nr	4
S Cho et al.	200	154PR 46SEC	nr	nr	nr	nr	nr	nr
Cafarotti et al.	1092	285PR 114SEC	97	324	272	nr	nr	nr
Messa et al.	44	nr	nr	nr	nr	nr	nr	nr
A Sale et al.	148	129PR 19SEC	nr	nr	nr	nr	nr	nr
Xianshuai Li et al.	677	nr	nr	677	nr	nr	nr	nr
Bauman et al.	189	nr	nr	nr	nr	nr	nr	nr
Keeling et al.	82 (93)	nr	63	8	22	nr	nr	nr
Kulvatunyou et al.	36	nr	nr	nr	nr	nr	nr	nr
Horsley et al.	44 (52)	5PR 9SEC	15	19	nr	nr	nr	4
Vedam et al.	36	36 SP	nr	nr	nr	nr	nr	nr
Liang et al.	133 (142)	nr	59	18	62	3TR	nr	nr
Mortman et al.	326	154	14	30	115	7	nr	5
B Jayakrishnan et al.	141	nr	nr	45	96	nr	nr	nr
C-H Chen et al.	168	168 SEC	nr	nr	nr	nr	nr	nr
Orlando et al.	160 (191)	nr	nr	nr	nr	160TR	nr	nr
Bauman et al.	20	nr	nr	nr	nr	20TR	nr	nr
Rafiq et al.	30	nr	22	6	nr	nr	2	nr
Cantin et al.	51	13	11	nr	nr	nr	26	1
Jain et al.	50	nr	43	4	nr	2	nr	1
Tajarernmuang et al.	205 (249)	5	81	nr	nr	12	nr	151

PR: primary, SEC: secondary, SP: spontaneous, TR: traumatic, nr: not reported.

**Table 6.** Outcomes of SBCDs.

**Study**	**No. of patients**	**Overall failure rate (%)**	**Second pigtail inserted**	**Chest tube inserted after failure**	**Failure requiring VATS**	**Surgical resections or thoracostomy**	**Patient died due to related reasons**	**Antibiotic prophylaxis**	**Comorbidities**	**Performing physician**
HE Davies et al.	91 (100)	30	13	nr	nr	1	nr	nr	nr	nr
Vetrugno et al.	71 (87)	19.3	17	16	1	nr	nr	nr	nr	Resident
MT Congedo et al.	117	15.4	nr	nr	nr	nr	nr	nr	nr	Consultant or resident
Kulvatunyou et al.	94	11.0	nr	1	nr	nr	nr	nr	nr	Attending or resident supervised by attending
Kulvatunyou et al.	20	5.0	nr	nr	nr	nr	nr	nr	nr	Attending or resident supervised by attending
Treml et al.	247 (396)	nr	nr	nr	nr	nr	nr	nr	nr	Experienced residents or consultants
Kulvatunyou et al.	56	11.0	1	nr	4	1	nr	No antibiotic prophylaxis was routinely given	nr	Attending or resident supervised by attending
Y-H Liu et al.	276 (332)	27.1	47	20	nr	nr	nr	nr	nr	Experienced pulmonologists
Y-C Lin et al.	62 (70)	31.4	nr	16	nr	3	nr	nr	Mentioned but no correlation	Experienced emergency physician or intensivist (also a pulmonologist)
Rivera et al.	107 (131)	14.1	4	2	4	nr	nr	Antibiotics were not routinely used, but many received prophylactic treatment for other procedures or injuries.	nr	Attending or resident supervised by attending
S Cho et al.	200	24.0	30	nr	18	nr	nr	nr	nr
Cafarotti et al.	1092	12.9	69	15	nr	nr	nr	Given to all patients	nr	nr
Messa et al.	44	30.0	15	nr	5	2	nr	nr	nr	nr
A Sale et al.	148	17.6	nr	nr	nr	9	nr	nr	nr	Emergency physician
Xianshuai Li et al.	677	nr	nr	Nr	nr	nr	nr	nr	nr	nr
Bauman et al.	189	21.0	2	8	7	1	nr	nr	nr	nr
Keeling et al.	82 (93)	20.4	8	3	nr	10	nr	nr	nr	Attending or resident supervised by attending
Kulvatunyou et al.	36	8.0	1	1	1	nr	nr	nr	nr	Attending or resident supervised by attending
Horsley et al.	44 (52)	37.0	8	6	nr	nr	nr	nr	nr	Attending
Vedam et al.	36	28.0	nr	6	nr	nr	nr	nr	nr	nr
Liang et al.	133 (142)	42.0	nr	nr	nr	nr	0	nr	nr	Attending
Mortman et al.	326	nr	8	nr	nr	nr	nr	nr	yes	Attending
B Jayakrishnan et al.	141	10.0	nr	nr	nr	nr	nr	nr	nr	nr
C-H Chen et al.	168	29.8	nr	34	27	nr	nr	nr	nr	Attending
Orlando et al.	160 (191)	4.2	8	nr	nr	nr	nr	nr	nr	nr
Bauman et al.	20	10	nr	nr	1	nr	nr	Antibiotics were not routinely administered	nr	Attending
Rafiq et al.	30	23	nr	nr	nr	nr	nr	nr	nr	nr
Cantin et al.	51	12	13	nr	nr	nr	nr	nr	Attending or resident supervised by attending
Jain et al.	50	8	nr	nr	nr	nr	nr	nr	nr	nr
Tajarernmuang et al.	205 (249)	21.5	44	nr	nr	nr	nr	nr	nr	Radiologist

nr: not reported.

**Table 7.** Pigtail catheter complications.

**Study**	**Number of patients (number of insertions)**	**Pneumothorax does not present before the procedure**	**Major hemorrhage**	**Minor bleeding/local bleeding**	**Infection/empyema**
HE Davies et al.	91 (100)	4	1	nr	nr
Vetrugno et al.	71 (87)	19	nr	nr	nr
Treml et al.	247 (396)	18	3	1	nr
Kulvatunyou et al.	56	nr	3	nr	nr
Y-H Liu et al.	276 (332)	nr	1	nr	5
Y-C Lin et al.	62 (70)	nr	nr	nr	3
Cafarotti et al.	1092	nr	nr	10	4
Messa et al.	44	nr	nr	nr	3
A Sale et al.	148	nr	nr	3
Xianshuai Li et al.	677	nr	nr	1	nr
Bauman et al.	189	nr	1	nr	nr
Keeling et al.	82 (93)	1	nr	nr	nr
Horsley et al.	44 (52)	2	nr	1	1
Vedam et al.	36	nr	nr	nr	nr
Liang et al.	133	nr	nr	nr	4
Mortman et al.	326	nr	nr	nr	nr
B Jayakrishnan et al.	141	4	nr	nr	nr
Orlando et al.	160 (191)	nr	nr	nr	2
Rafiq et al.	30	1	nr	nr	nr
Jain et al.	50	10	nr	nr	nr
Tajarernmuang et al.	205 (249)	55	nr	2	nr

nr: not reported.

**Table 8.** Insertion related complications.

**Study**	**Number of patients (number of insertions)**	**Image-guided**	**Tube dislodgment**	**Tube kinking**	**Drain blockage**	**Unsuccessful insertion attempt**	**Misplacement/malposition**	**Iatrogenic organ injury**	**Subcutaneous hematoma**
HE Davies et al.	91 (100)	Yes	21	nr	9	1	nr	nr	nr
Vetrugno et al.	71 (87)	Yes	28	8	nr	1	18	nr	nr
MT Congedo et al.	117	No	16	nr	3	nr	nr	nr	nr
Kulvatunyou et al.	94	No	nr	nr	nr	nr	1	nr	nr
Kulvatunyou et al.	20	No	1	nr	nr	1	nr	nr	nr
Treml et al	247 (396)	No	nr	nr	nr	nr	1	nr	nr
Kulvatunyou et al.	56	Yes	nr	nr	nr	nr	1	nr	nr
Y-H Liu et al.	276 (332)	Yes	4	nr	nr	nr	nr	1	1
Y-C Lin et al.	62 (70)	Yes	nr	nr	1	nr	nr	nr	nr
Rivera et al.	107 (131)	Yes	2	nr	nr	nr	1	nr	nr
S Cho et al.	200	No	2	3	nr	nr	nr	nr	nr
Cafarotti et al.	1092	No	59	nr	82	nr	nr	nr	nr
Messa et al.	44	No	nr	nr	nr	nr	2	nr	nr
Xianshuai Li et al.	677	No	nr	nr	3	nr	nr	nr	1
Bauman et al.	189	No	4	nr	nr	nr	2	2	nr
Keeling et al.	82(93)	Yes	12	nr	5	11	1	nr	1
Kulvatunyou et al.	36	No	1	nr	nr	nr	2	nr	nr
Horsley et al.	44 (52)	No	4	nr	12	nr	nr	nr	nr
Vedam et al.	36	No	2	nr	5	nr	nr	nr	nr
Liang et al.	133 (142)	Yes	1	7	nr	nr	nr	nr	3
Mortman et al.	326	Yes	nr	nr	nr	nr	12	3	nr
B Jayakrishnan et al.	141	Yes	nr	nr	4	nr	nr	nr	nr
Orlando et al.	160 (191)	No	nr	nr	8	nr	7	nr	nr
Rafiq et al.	30	Yes	1	1	2	nr	nr	nr	nr
Jain et al.	50	No	nr	nr	4	nr	nr	nr	nr
Tajarernmuang et al.	205 (249)	Yes	nr	7	18	nr	nr	3	nr

nr: not reported.
